# Preoperative CT-Based Pelvic Sarcopenia and Subcutaneous Adiposity Are Associated with Anaemia and Operative Time in Acetabular Fracture Surgery: A Retrospective Cohort Study

**DOI:** 10.3390/medicina62061036

**Published:** 2026-05-26

**Authors:** Kürşat Tuğrul Okur, Ferid Abdulaliyev, Süleyman Yalçın, Eda İştahlı, Mustafa İştahlı, Ali Koç, Fırat Ozan

**Affiliations:** 1 Department of Orthopaedics and Traumatology, Health Sciences University Trabzon Kanuni Training and Research Hospital, 61290 Trabzon, Türkiye; 2 Department of Orthopaedics and Traumatology, Yozgat Sorgun State Hospital, 66700 Yozgat, Türkiye; 3 Department of Orthopaedics and Traumatology, University of Health Sciences, Kayseri City Training and Research Hospital, 38080 Kayseri, Türkiye; 4 Department of Radiology, University of Health Sciences, Kayseri City Training and Research Hospital, 38080 Kayseri, Türkiye

**Keywords:** acetabular fracture, sarcopenia, subcutaneous adipose tissue, cross-sectional area, opportunistic CT screening, operative time, haemoglobin, orthopaedic trauma

## Abstract

*Background and Objectives*: Acetabular fracture surgery is associated with substantial perioperative blood loss and prolonged operative time. Routine preoperative pelvic computed tomography (CT) carries information about body composition that is not currently exploited for risk stratification. We tested whether (i) CT-defined pelvic sarcopenia is associated with lower preoperative haemoglobin and (ii) preoperative subcutaneous fat cross-sectional area (CSA) is independently associated with operative time, after adjustment for surgical approach, age, fracture complexity and sarcopenia status. *Materials and Methods*: In this single-centre retrospective cohort study, 48 adults (37 men, 11 women; mean age 40.2 ± 16.5 years) who underwent open reduction and internal fixation (ORIF) for unilateral acetabular fractures between 2016 and 2024 were included. Pelvic muscle and subcutaneous fat CSAs were measured on the contralateral side of preoperative CT images using ImageJ. Sarcopenia was defined as an internal, cohort-relative classification based on the sex-specific bottom tertile of psoas CSA. Normality was assessed by Shapiro–Wilk testing; Pearson or Spearman correlation was used accordingly, and the 36 pairwise correlations were controlled with the Benjamini–Hochberg false-discovery-rate procedure. The multivariable model used ordinary least squares regression with heteroscedasticity-consistent (HC3) standard errors and a median quantile-regression robustness check. *Results*: Sarcopenic patients (n = 17) had significantly lower preoperative haemoglobin (12.63 ± 1.24 vs. 14.00 ± 1.53 g/dL; *p* = 0.002; Cohen’s d = 0.96). The absolute perioperative haemoglobin drop was numerically smaller in the sarcopenic group (ΔHb 1.64 ± 0.91 vs. 2.46 ± 1.87 g/dL) but did not reach statistical significance (*p* = 0.079); estimated blood loss (*p* = 0.258) and transfusion requirement (*p* = 0.567) did not differ between groups. Pelvic muscle CSAs correlated positively with preoperative haemoglobin (all q < 0.05 after Benjamini–Hochberg correction). In the multivariable model (F[6, 41] = 3.71, *p* = 0.005; adjusted R^2^ = 0.26; all variance inflation factors 1.06–1.26), subcutaneous fat CSA (B = +0.25 min/cm^2^, *p* = 0.004) and the modified Stoppa approach (vs. Kocher–Langenbeck; +65 min, *p* = 0.001) were independently associated with operative time. *Conclusions*: In this exploratory retrospective cohort, routine preoperative pelvic CT contained two body-composition signals that may warrant prospective evaluation: pelvic sarcopenia, which was associated with lower baseline haemoglobin, and subcutaneous adiposity, which was associated with longer operative time in the primary regression model. Both signals require confirmation—the sarcopenia–bleeding relationship was not statistically significant, and the subcutaneous fat association was attenuated under robust inference. These findings are hypothesis-generating; prospective multicentre validation with height-normalised sarcopenia thresholds and body mass index is required before clinical implementation.

## 1. Introduction

Acetabular fractures are among the most demanding injuries in orthopaedic trauma surgery. Most are caused by high-energy mechanisms, frequently in patients with concomitant injuries, and surgical reconstruction often demands long operative times, anatomically deep dissection, and considerable intraoperative blood loss [1,2,3,4]. Anterior intrapelvic approaches that permit early definitive fixation are increasingly used and have been shown to be safe in appropriately selected patients [5]. The pelvic ring is highly vascular, and uncontrolled haemorrhage remains a leading cause of perioperative morbidity and mortality; transfusion requirement, length of hospital stay, and operative duration each contribute meaningfully to overall outcome, with intrapelvic vascular injuries such as superior gluteal artery disruption representing a particularly high-risk scenario [6,7,8,9,10].

Risk stratification before acetabular fracture surgery currently relies on a small set of variables: age, fracture pattern, mechanism of injury, body mass index, and preoperative laboratory values, principally haemoglobin (Hb). Yet every patient who reaches the operating room for acetabular ORIF has already had a preoperative pelvic computed tomography (CT) scan for surgical planning. That image contains a great deal of information about the patient’s body composition that is presently discarded: the cross-sectional areas (CSAs) of pelvic skeletal muscles, the radiodensity of those muscles, and the thickness of subcutaneous adipose tissue [11,12,13,14,15].

Two strands of the literature support the value of extracting these parameters. First, low psoas CSA on abdominopelvic CT is an accepted opportunistic surrogate for sarcopenia and has been linked to postoperative complications, prolonged length of stay, and mortality across general surgical, hip-fracture, and trauma populations [12,14,15,16]. We note that much of this supporting literature derives from geriatric, hip-fracture, or non-orthopaedic surgical populations. Within orthopaedic trauma populations, studies investigating the impact of body composition on perioperative outcomes remain scarce. Second, in pelvic and acetabular trauma, body habitus and local soft-tissue thickness are known to influence operative complexity, but the literature has chiefly relied on body mass index—a composite measure that aggregates muscle, visceral adiposity, and subcutaneous adiposity into a single value and obscures their individual contributions [8,9,17]. CT-based, anatomically specific measurements offer a more direct mechanical correlate.

This study therefore tested two prespecified hypotheses in a retrospective cohort of patients undergoing ORIF for unilateral acetabular fractures. First, that pelvic sarcopenia would be associated with lower preoperative haemoglobin. Second, that preoperative subcutaneous fat CSA would be independently associated with operative time.

## 2. Materials and Methods

### 2.1. Study Design and Participants

This single-centre retrospective cohort study was conducted at a tertiary referral hospital. The hospital surgical record system was searched for all adult patients (≥18 years) who underwent ORIF for an acetabular fracture between January 2016 and December 2024. From an initial search yielding 76 patients, 48 met the inclusion criteria of (i) unilateral acetabular fracture, (ii) preoperative pelvic CT available in DICOM format, and (iii) complete intraoperative and perioperative records. Patients with bilateral acetabular fractures, pathological fractures, prior pelvic surgery on the contralateral side, or incomplete imaging or laboratory data were excluded. The study followed the STROBE statement. The study was conducted in accordance with the Declaration of Helsinki and was approved by the University of Health Sciences Kayseri City Training and Research Hospital Clinical Research Ethics Committee (approval date: 27 May 2025; approval number: 460). Informed consent was waived owing to the retrospective nature of the analysis.

### 2.2. Clinical and Demographic Data

Age, sex, mechanism of injury, length of hospital stay, length of intensive care unit (ICU) stay, presence and type of concomitant injuries (defined as polytrauma when any other anatomical region was injured), neurological deficit, and preoperative waiting time were extracted from the electronic medical record. Fractures were classified using the Letournel–Judet system from the operative report and preoperative CT and were grouped into elementary and complex/associated patterns [18]. Surgical approach (Kocher–Langenbeck, modified Stoppa, ilioinguinal) was recorded as documented by the operating surgeon. Operative time was extracted from the anaesthesia record. Preoperative haemoglobin was the most recent value within 24 h before surgery; postoperative haemoglobin was the value on postoperative day 1. ΔHb was calculated as preoperative minus postoperative. Estimated total blood loss was recorded from the anaesthesia chart, and red-cell transfusion as the number of units of packed red blood cells administered between admission and 48 h postoperatively.

### 2.3. CT-Based Body-Composition Measurement

**Figure 1:**
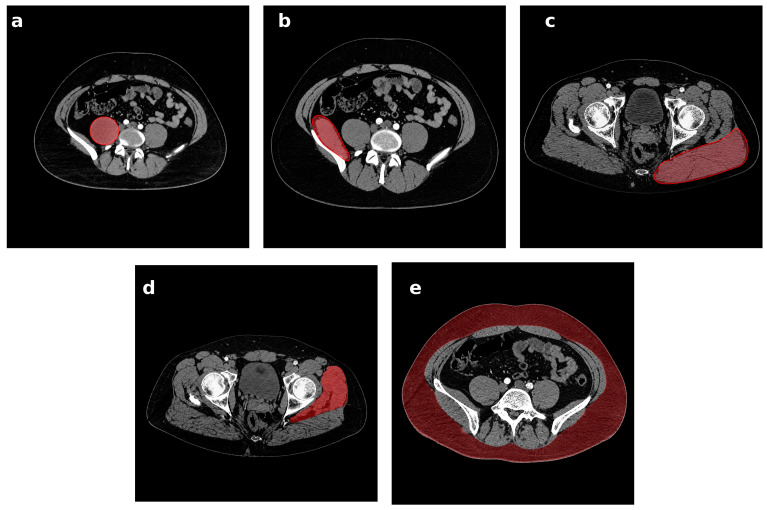
Representative axial computed tomography images showing the standardised anatomical levels at which pelvic body-composition measurements were obtained on the contralateral (uninjured) hemipelvis. The segmented region of interest for each structure is shaded in red. (**a**) Psoas major at the L4–L5 intervertebral level; (**b**) iliacus at the L4–L5 intervertebral level; (**c**) gluteus maximus at the level of the centre of the acetabulum; (**d**) gluteus medius–minimus at the level of the centre of the acetabulum; (**e**) subcutaneous adipose tissue at the L5 vertebral body level.

All measurements were performed on preoperative pelvic CT acquired on a 64-slice multidetector scanner (slice thickness 1 mm). To eliminate the effect of trauma-induced soft-tissue change, all measurements were taken on the contralateral (uninjured) hemipelvis. Manual segmentation was performed in ImageJ (version 1.54). Cross-sectional areas (cm^2^) were measured at standardised anatomical levels: psoas major and iliacus at the L4–L5 intervertebral level; gluteus maximus and gluteus medius–minimus at the level of the centre of the acetabulum; subcutaneous adipose tissue at the L5 vertebral level, between the skin and the lateral fascial border (Figure 1). Muscle radiodensity was recorded in Hounsfield units (HUs) as a surrogate of muscle quality. Segmentations were performed jointly by orthopaedic surgeons and radiologists, with 30 randomly selected images re-segmented after a four-week washout for intra-rater reliability and 20 images independently segmented by a second observer for inter-rater reliability. The intraclass correlation coefficient for CSA measurements was 0.95.

### 2.4. Sarcopenia Definition

Sarcopenia was defined a priori as a psoas CSA in the bottom tertile of the sex-specific distribution within the cohort (sex-specific cut-offs: 12.90 cm^2^ for men and 8.08 cm^2^ for women). As a sensitivity analysis, sarcopenia was redefined using a sex-specific median split.

### 2.5. Statistical Analysis

Continuous variables are reported as mean ± standard deviation and categorical variables as count (percentage). The distribution of each continuous variable was assessed by visual inspection of histograms and normal quantile–quantile (Q–Q) plots, supported by the Shapiro–Wilk test; because the Shapiro–Wilk test has limited power in samples of this size, the graphical assessment was treated as the primary basis for the parametric-versus-non-parametric decision and the Shapiro–Wilk result as confirmatory. On this combined assessment, preoperative haemoglobin, postoperative haemoglobin and all pelvic muscle cross-sectional areas were approximately normally distributed, whereas Δ haemoglobin, estimated blood loss, transfusion requirement, operative time, length of stay and subcutaneous fat cross-sectional area showed appreciable departures from normality. Between-group comparisons used Welch’s *t*-test for approximately normally distributed variables and the Mann–Whitney U test otherwise; categorical variables were compared with Fisher’s exact test. Effect sizes are reported as Cohen’s d. Pairwise associations between body-composition parameters and perioperative outcomes were assessed with Pearson’s correlation when both variables were approximately normally distributed and Spearman’s rank correlation otherwise; the coefficient type for each cell of Table 1 is indicated in the table footnote. Because Table 1 comprises 36 pairwise tests, raw *p*-values were adjusted for multiple comparisons using the Benjamini–Hochberg false-discovery-rate procedure, and both raw *p*-values and adjusted q-values are reported.

Independent predictors of operative time were examined using a multivariable ordinary least squares (OLS) linear regression model with six predictors: subcutaneous fat cross-sectional area, surgical approach (entered as two indicator variables, with Kocher–Langenbeck as the reference category), age, fracture complexity (associated versus elementary Letournel–Judet pattern), and sarcopenia status. Body mass index could not be entered into the model because patient height was not consistently documented. With 48 observations and six predictors, the subjects-to-predictor ratio was 8, below the commonly recommended minimum of 10–15 observations per predictor for stable coefficient estimation in linear regression; the regression results are therefore presented as exploratory, and coefficient estimates should be regarded as provisional and potentially unstable under resampling. Model adequacy was assessed using variance inflation factors (VIFs) for multicollinearity, the Durbin–Watson statistic for residual autocorrelation, the Shapiro–Wilk test of the regression residuals for normality, and the Breusch–Pagan test for heteroscedasticity. Because the residual diagnostics indicated departures from the classical OLS assumptions, the model was re-estimated with heteroscedasticity-consistent (HC3) standard errors as the principal sensitivity analysis; a median (0.5-quantile) regression was additionally fitted as a non-parametric robustness check, and a predictor was considered robust only where its statistical significance was concordant across these analyses. A two-sided *p* < 0.05 was considered statistically significant. As data collection had concluded at the time of analysis, an a priori sample size calculation was not appropriate; instead, a sensitivity (post hoc) power analysis was performed at two-sided α = 0.05 and 80% power. Given the sample size, the study could reliably detect only a Pearson correlation of |r| ≥ 0.40, a between-group standardised difference of Cohen’s d ≥ 0.86 for the primary sarcopenia comparison (n = 17 versus 31), and a regression effect size of Cohen’s f^2^ ≥ 0.33; weaker associations cannot be excluded, and non-significant findings are therefore interpreted as inconclusive rather than as evidence of no effect. Analyses were performed in Python 3.12 (statsmodels 0.14, SciPy 1.13, pandas 2.2).

## 3. Results

### 3.1. Cohort Characteristics

Of 48 included patients, 37 (77.1%) were men. The mean age was 40.2 ± 16.5 years (range 19–72). Mechanisms of injury were traffic accidents in 33 (68.8%) patients, falls from height in 14 (29.2%), and direct trauma in 1 (2.1%). Furthermore, 20 patients (41.7%) had at least one concomitant injury (polytrauma), and 14 (29.2%) had a complex/associated fracture pattern. The Kocher–Langenbeck approach was used in 28 patients (58.3%), the modified Stoppa approach in 14 (29.2%), and the ilioinguinal approach in 6 (12.5%). Mean operative time was 155.5 ± 61.6 min and mean estimated total blood loss was 1542.5 ± 862.5 mL. Mean preoperative Hb was 13.5 ± 1.6 g/dL, mean postoperative day-1 Hb 11.4 ± 1.2 g/dL, and mean ΔHb 2.2 ± 1.6 g/dL.

Within the 20 polytrauma patients, documented concomitant injury patterns were urological injuries requiring repair (bladder or urethral injury; n = 4), maxillofacial or ocular injuries requiring intervention (n = 3), concomitant long-bone fractures (n = 6), and thoracoabdominal injuries (n = 6); several patients had more than one of these. Cohort characteristics, overall and stratified by sarcopenia status, are summarised in Table 2.

### 3.2. Pelvic Sarcopenia Is Associated with Lower Preoperative Haemoglobin

**Figure 2:**
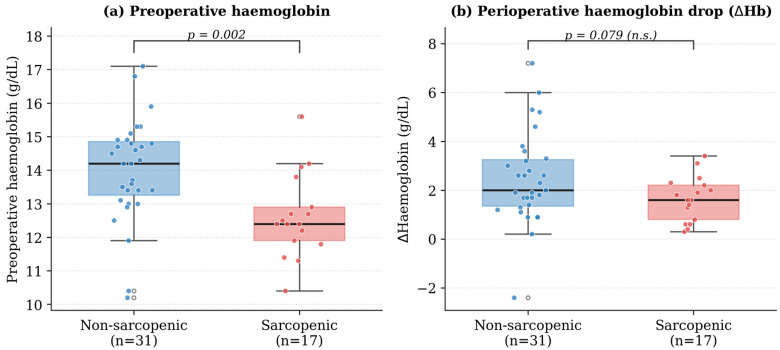
Preoperative haemoglobin and perioperative haemoglobin drop, by sarcopenia status. (**a**) Preoperative haemoglobin was significantly lower in sarcopenic patients (12.63 ± 1.24 vs. 14.00 ± 1.53 g/dL; Welch’s *t*-test, *p* = 0.002). (**b**) The perioperative haemoglobin drop was numerically smaller in sarcopenic patients (1.64 ± 0.91 vs. 2.46 ± 1.87 g/dL) but did not differ significantly between groups (Mann–Whitney U test, *p* = 0.079). Boxes show the median and interquartile range; whiskers show 1.5× IQR; individual patients are overlaid as points.

By the sex-specific bottom-tertile definition, 17 of 48 patients (35.4%) were classified as sarcopenic. Sarcopenic and non-sarcopenic groups did not differ significantly in age, sex, polytrauma rate, fracture complexity, or surgical approach (Table 2). Sarcopenic patients had significantly lower preoperative haemoglobin (12.63 ± 1.24 vs. 14.00 ± 1.53 g/dL; mean difference −1.37 g/dL, 95% CI −2.20 to −0.55; *p* = 0.002; Cohen’s d = 0.96). The absolute perioperative haemoglobin drop was numerically smaller in the sarcopenic group (ΔHb 1.64 ± 0.91 vs. 2.46 ± 1.87 g/dL), but this difference did not reach statistical significance (Mann–Whitney U test, *p* = 0.079; Cohen’s d = 0.52); the direction is nonetheless consistent with the lower starting baseline in sarcopenic patients (Figure 2). Estimated total blood loss (*p* = 0.258) and red-cell transfusion requirement (*p* = 0.567) also did not differ significantly between groups (Table 3). Taken together, these results indicate that pelvic sarcopenia is associated with lower baseline haemoglobin but provide no evidence that it is associated with greater perioperative blood loss; given the limited statistical power, however, a modest difference in perioperative bleeding cannot be excluded. Operative time and ICU stay did not differ significantly between groups.

Length of hospital stay was numerically shorter in the sarcopenic group (8.71 ± 4.48 vs. 11.97 ± 5.76 days; Mann–Whitney U test, *p* = 0.017). On re-examination, this was not explained by between-group differences in polytrauma frequency (47.1% vs. 38.7%; Fisher’s exact *p* = 0.760), and the pattern persisted within both polytrauma strata (non-polytrauma: 7.6 ± 1.8 vs. 11.3 ± 5.9 days; polytrauma: 10.0 ± 6.2 vs. 13.0 ± 5.6 days). Only three patients (6.3%) had a stay exceeding 21 days—one polytrauma patient with bladder rupture and maxillofacial fractures (29 days, ICU 12 days) and two patients with isolated acetabular fractures (23 days, ICU 0; and 22 days, ICU 7); all three were non-sarcopenic.

In the sensitivity analysis using a sex-specific median split (n = 22 sarcopenic vs. 26 non-sarcopenic), the direction and magnitude of the haemoglobin difference were preserved (preoperative Hb 12.93 ± 1.45 vs. 14.02 ± 1.51 g/dL; *p* = 0.015; d = 0.73), supporting the robustness of the primary finding to the sarcopenia definition.

### 3.3. Body Composition Correlates with Preoperative Haemoglobin

Pelvic muscle CSA and preoperative haemoglobin showed consistent positive correlations: psoas CSA (r = +0.49, *p* < 0.001), iliacus CSA (r = +0.42, *p* = 0.003), gluteus medius–minimus CSA (r = +0.44, *p* = 0.002), and total muscle CSA (r = +0.44, *p* = 0.002). After Benjamini–Hochberg adjustment of the 36 pairwise correlations at a false-discovery rate of 0.05, these four associations between pelvic muscle CSAs and preoperative haemoglobin remained statistically significant (all q < 0.05); the gluteus maximus–transfusion correlation was a fifth surviving association (Spearman ρ = −0.39, q = 0.043). The unadjusted subcutaneous fat CSA–operative time correlation (Spearman ρ = +0.31, raw *p* = 0.033) did not survive multiplicity correction (q = 0.149); the association between subcutaneous fat CSA and operative time is therefore reported on the basis of the multivariable regression model rather than the unadjusted bivariate correlation. The full correlation matrix is provided in Table 1.

### 3.4. Subcutaneous Fat CSA Is Independently Associated with Operative Time

**Figure 3:**
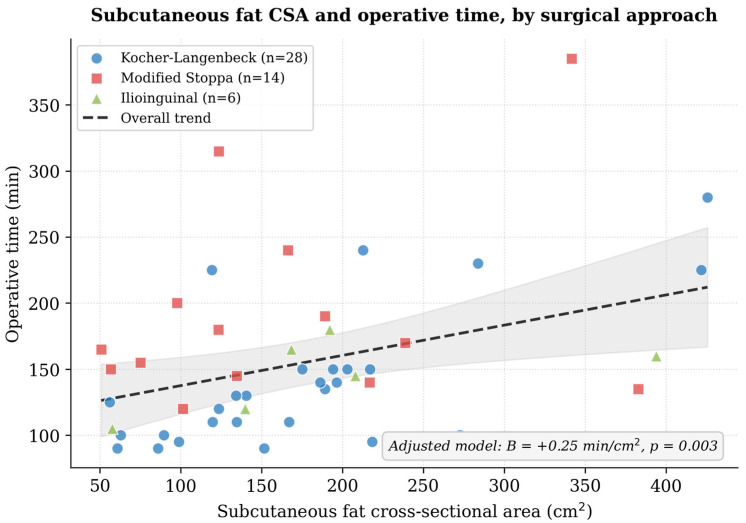
Relationship between preoperative subcutaneous fat cross-sectional area (CSA) and operative time, stratified by surgical approach. Each point represents one patient; the dashed line shows the overall linear trend with its 95% confidence band. In the multivariable model adjusting for surgical approach, age, fracture complexity and sarcopenia status, subcutaneous fat CSA was independently associated with operative time (B = +0.25 min/cm^2^; *p* = 0.004); under heteroscedasticity-consistent (HC3) inference the association was attenuated to borderline significance (*p* = 0.068). The unadjusted bivariate correlation (Spearman ρ = +0.31, raw *p* = 0.033) did not survive Benjamini–Hochberg multiplicity correction (q = 0.149).

**Figure 4:**
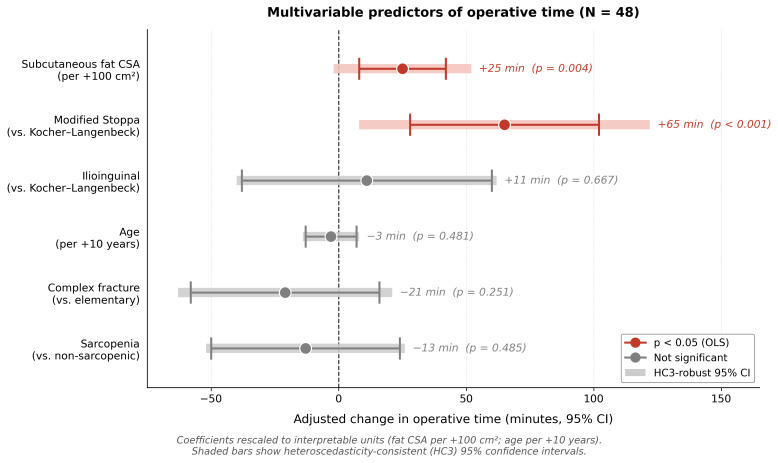
Multivariable linear regression for operative time (N = 48). Points show the adjusted change in operative time associated with each predictor, with coefficients rescaled to interpretable units (subcutaneous fat CSA per +100 cm^2^; age per +10 years) for cross-predictor comparability. Solid error bars show ordinary least squares 95% confidence intervals; shaded bars show heteroscedasticity-consistent (HC3) 95% confidence intervals, added as a sensitivity analysis. Red denotes predictors significant at *p* < 0.05 in the ordinary least squares model. The modified Stoppa approach was the strongest contributor to operative time; subcutaneous fat CSA was an additional, statistically independent contributor of smaller absolute magnitude. The ilioinguinal approach, age, fracture complexity and sarcopenia status were not significantly associated with operative time.

The multivariable OLS regression model for operative time was statistically significant overall (F[6, 41] = 3.71, *p* = 0.005) and explained a modest proportion of the variance (R^2^ = 0.352; adjusted R^2^ = 0.257), indicating that most of the variation in operative time was not captured by the measured predictors. Multicollinearity was negligible: all variance inflation factors were between 1.06 and 1.26 (subcutaneous fat CSA 1.10; modified Stoppa 1.17; ilioinguinal 1.11; age 1.06; fracture complexity 1.18; sarcopenia 1.26). Two predictors were independently and significantly associated with operative time. Each 1 cm^2^ increase in subcutaneous fat CSA was associated with a 0.25 min increase in operative time (B = +0.25 min/cm^2^, 95% CI +0.09 to +0.41; *p* = 0.004), independent of surgical approach, age, fracture complexity, and sarcopenia (Figure 3). The modified Stoppa approach was associated with operative time approximately 65 min longer than the Kocher–Langenbeck approach (B = +65.4 min, 95% CI +28.6 to +102.3; *p* = 0.001). The ilioinguinal approach (B = +10.6 min; *p* = 0.667), age (B = −0.34 min/year; *p* = 0.481), fracture complexity (B = −21.4 min; *p* = 0.251) and sarcopenia status (B = −12.7 min; *p* = 0.485) were not independently associated with operative time. Full model results are presented in Table 4, and the contributions of each predictor are summarised in Figure 4.

Regression diagnostics indicated departures from the classical OLS assumptions: the Shapiro–Wilk test of the residuals (*p* = 0.006) suggested non-normal residuals and the Breusch–Pagan test (*p* = 0.004) indicated heteroscedasticity, whereas the Durbin–Watson statistic (1.50) was consistent with the absence of substantial residual autocorrelation. The model was therefore re-estimated with heteroscedasticity-consistent (HC3) standard errors. Under HC3 inference, the modified Stoppa approach remained significantly associated with operative time (B = +65.4 min, 95% CI +8.6 to +122.3; *p* = 0.024), whereas the association for subcutaneous fat CSA was attenuated to borderline significance (B = +0.25 min/cm^2^, 95% CI −0.02 to +0.52; *p* = 0.068). In an additional median quantile-regression sensitivity analysis, both the modified Stoppa approach (B = +64.0 min; *p* < 0.001) and subcutaneous fat CSA (B = +0.34 min/cm^2^; *p* < 0.001) were significantly associated with operative time. The association of subcutaneous fat CSA with operative time was thus significant in the OLS and quantile-regression models but only borderline under HC3-robust inference; we therefore regard it as a suggestive but not definitively robust finding. The association of surgical approach with operative time was concordant and significant across all three analyses. We emphasise that the absolute contribution of subcutaneous fat CSA is modest; the explanatory power of the model is limited (adjusted R^2^ = 0.26), and all coefficient estimates should be regarded as exploratory.

## 4. Discussion

In this exploratory retrospective cohort of 48 patients undergoing ORIF for unilateral acetabular fractures, two body-composition signals derived at no marginal cost from routine preoperative pelvic CT were associated with distinct perioperative parameters. First, pelvic sarcopenia—defined as a sex-specific bottom-tertile psoas CSA—was associated with markedly lower preoperative haemoglobin (mean difference 1.4 g/dL). The absolute perioperative haemoglobin drop was numerically smaller in sarcopenic patients but did not differ significantly between groups, and neither estimated blood loss nor transfusion requirement differed; we therefore interpret pelvic sarcopenia as a marker of lower baseline haematological reserve rather than of increased intraoperative bleeding. Second, after adjustment for surgical approach, age, fracture complexity, and sarcopenia status, subcutaneous fat CSA was independently associated with operative time in the primary OLS model. Surgical approach was the single strongest contributor: a modified Stoppa procedure was associated with an operative time approximately 65 min longer than a Kocher–Langenbeck procedure, consistent with the known mechanical and exposure differences between anterior intrapelvic and posterior approaches. Against this dominant background, subcutaneous fat CSA emerged as an additional contributor to operative time (OLS B = +0.25 min/cm^2^), suggesting that local soft-tissue characteristics may influence surgical complexity beyond the effect of the chosen approach.

The dominant contribution of surgical approach to operative time is consistent with recent higher-level evidence [19,20,21,22,23,24]. A 2025 systematic review and network meta-analysis by Ramadanov and colleagues directly compared pararectus, ilioinguinal, and intrapelvic approaches for acetabular fractures and ranked them with respect to operative efficiency and intraoperative blood loss, demonstrating clinically meaningful differences between approaches [25]. The approximately 65 min difference between the modified Stoppa and Kocher–Langenbeck approaches observed here is in keeping with the magnitude of approach-related differences reported in that network meta-analysis and in earlier pairwise meta-analytic comparisons [26]. These data reinforce that the surgical approach is the principal determinant of operative time, against which patient-level body-composition parameters act as secondary, additive covariates.

Our findings extend the growing literature on opportunistic CT-based sarcopenia assessment. Wang and colleagues reported that low muscle size and density were independently associated with mortality after hip fracture [14]. Nishimura and colleagues identified the psoas muscle index as a predictor of mortality and morbidity in geriatric trauma patients [16]. We acknowledge that much of this supporting literature derives from geriatric, hip-fracture, or non-orthopaedic surgical populations and is therefore not a direct comparator for the relatively young acetabular trauma cohort studied here (mean age 40 years); these studies establish the mechanistic rationale rather than equivalence of clinical context. The applicability of our findings to geriatric acetabular fracture populations remains an explicit open question for prospective work.

The association between subcutaneous fat CSA and operative time observed in the primary OLS model is directionally consistent with prior observations that obesity influences operative complexity in pelvic and acetabular trauma surgery [8,9,17]. CT-derived subcutaneous fat CSA may be a more direct mechanical correlate of soft-tissue dissection depth than BMI, but the present study cannot demonstrate the incremental value of CSA over BMI because BMI could not be computed in this cohort. The proposition that CSA outperforms BMI as a predictor of operative time should be tested directly in prospective cohorts with both measurements available.

There are several limitations. First, the retrospective single-centre design limits generalisability and precludes adjustment for unmeasured confounders. Body mass index could not be included in the multivariable model because height was not consistently recorded; consequently, we cannot determine whether CT-derived subcutaneous fat CSA provides information beyond a simple BMI measurement, and the incremental-value hypothesis remains untested. Second, with 48 observations and six predictors the subjects-to-predictor ratio was 8, below the conventional minimum of 10–15; the adjusted R^2^ of 0.26 confirms that a substantial proportion of variability in operative time remains unexplained, and coefficient estimates should be regarded as exploratory and potentially unstable under resampling. The sample gave 80% power to detect only Cohen’s d ≥ 0.86, |r| ≥ 0.40, and Cohen’s f^2^ ≥ 0.33; weaker effects cannot be excluded, and the non-significant comparisons—including the perioperative haemoglobin drop, estimated blood loss and transfusion requirement—should therefore be interpreted as inconclusive rather than as evidence of no effect. Third, two-dimensional CSA was used as a surrogate for muscle and adipose mass. Fourth, sarcopenia was defined relative to the cohort’s sex-specific distribution rather than against an externally validated, height-normalised threshold; the resulting classification is internal to this study and not directly comparable to published categories. Fifth, mean cohort age was lower than is typical of the sarcopenia literature. Sixth, the precise number of additional non-orthopaedic operative interventions could not be reliably extracted from the retrospective records. In view of these limitations, the present results should be interpreted as exploratory; the associations we report require independent confirmation in a larger, prospective, multicentre cohort before they can support clinical decision-making.

The potential clinical implications are practical but require prospective validation. The same preoperative pelvic CT obtained for surgical planning may, in future prospective work, be evaluable as an opportunistic source of two body-composition signals: psoas CSA at the L4–L5 level as a candidate marker of haematopoietic reserve and subcutaneous fat CSA at the L5 level as a candidate covariate for operative-time prediction. Because the segmentation can be completed in under five minutes with excellent reliability (ICC 0.95) and adds neither cost nor radiation exposure, the marginal effort is small. Until prospective multicentre validation is available, our findings should be regarded as hypothesis-generating, and we do not advocate routine clinical implementation of CT-based pelvic body-composition phenotyping in acetabular fracture surgery on the basis of the present data alone.

## 5. Conclusions

In this exploratory retrospective cohort, routine preoperative pelvic CT in acetabular fracture surgery contained two body-composition signals with distinct perioperative associations. Pelvic sarcopenia, defined as bottom-tertile psoas CSA, was associated with significantly lower preoperative haemoglobin and may serve as an opportunistic preoperative marker of lower baseline haematological reserve. The perioperative haemoglobin drop, estimated blood loss and transfusion requirement did not differ significantly between sarcopenic and non-sarcopenic patients; we therefore found no evidence that sarcopenia is associated with increased surgical bleeding, although the limited statistical power means a modest difference cannot be excluded. Subcutaneous fat CSA was independently associated with operative time in the primary regression model, but this association was attenuated to borderline significance under heteroscedasticity-consistent inference and should be regarded as suggestive rather than definitively established; surgical approach was the single strongest and most robust determinant of operative time. These findings are exploratory and hypothesis-generating. Validation in a larger, multicentre prospective cohort, with prospectively recorded height to enable externally validated height-normalised sarcopenia thresholds, body mass index, and functional outcome measures, is required before the proposed opportunistic CT-based phenotyping can be recommended for clinical use.

## Figures and Tables

**Table 1:** Correlations between pelvic body-composition parameters and perioperative outcomes (N = 48).

Body Composition	Preop Hb	Postop Hb	ΔHb	Blood Loss	Transfusion	Op. Time
Psoas CSA	+0.49 ***	+0.21	+0.35	+0.15	−0.07	−0.06
Iliacus CSA	+0.42 **	+0.21	+0.23	+0.03	−0.14	+0.14
Gluteus med–min CSA	+0.44 **	+0.21	+0.22	+0.05	−0.20	+0.15
Gluteus maximus CSA	+0.23	+0.19	+0.05	−0.22	−0.39 *	+0.10
Total muscle CSA	+0.44 **	+0.25	+0.22	−0.06	−0.32	+0.09
Subcutaneous fat CSA	+0.18	−0.07	+0.16	+0.17	+0.12	+0.31

Pearson’s correlation was used when both variables were approximately normally distributed, Spearman’s rank correlation otherwise. After Benjamini–Hochberg adjustment at q = 0.05 across all 36 cells: * q < 0.05; ** q < 0.05 and raw *p* < 0.01; *** q < 0.05 and raw *p* < 0.001. Hb: haemoglobin; CSA: cross-sectional area.

**Table 2:** Cohort characteristics, overall and stratified by sarcopenia status.

Variable	Overall (n = 48)	Non-Sarcopenic (n = 31)	Sarcopenic (n = 17)	*p*
Age (years)	40.2 ± 16.5	38.7 ± 17.6	43.1 ± 14.5	0.326
Male sex, n (%)	37 (77.1)	24 (77.4)	13 (76.5)	1.000
Polytrauma, n (%)	20 (41.7)	12 (38.7)	8 (47.1)	0.760
Complex fracture, n (%)	14 (29.2)	12 (38.7)	2 (11.8)	0.095
Kocher–Langenbeck, n (%)	28 (58.3)	20 (64.5)	8 (47.1)	0.522
Modified Stoppa, n (%)	14 (29.2)	8 (25.8)	6 (35.3)	–
Ilioinguinal, n (%)	6 (12.5)	3 (9.7)	3 (17.6)	–
Psoas CSA (cm^2^)	13.2 ± 3.9	15.0 ± 3.5	9.9 ± 2.4	<0.001
Total muscle CSA (cm^2^)	106.5 ± 19.0	110.8 ± 18.2	98.6 ± 18.3	0.033
Subcutaneous fat CSA (cm^2^)	178.7 ± 99.8	196.7 ± 106.0	146.0 ± 80.2	0.093

Values are mean ± SD or n (%). *p*-values from Welch’s *t*-test or Mann–Whitney U for continuous variables and Fisher’s exact test for categorical variables. Sarcopenia defined as sex-specific bottom-tertile psoas CSA. CSA: cross-sectional area.

**Table 3:** Perioperative outcomes by sarcopenia status.

Outcome	Non-Sarcopenic (n = 31)	Sarcopenic (n = 17)	*p*	Test
Preoperative haemoglobin (g/dL)	14.00 ± 1.53	12.63 ± 1.24	0.002	Welch
Postoperative day-1 Hb (g/dL)	11.54 ± 1.15	10.99 ± 1.09	0.110	Welch
Δ Haemoglobin (g/dL)	2.46 ± 1.87	1.64 ± 0.91	0.079	MWU
Estimated total blood loss (mL)	1668.4 ± 960.1	1312.9 ± 608.5	0.258	MWU
Transfusion (units of pRBC)	1.71 ± 1.49	1.65 ± 1.69	0.567	MWU
Operative time (min)	160.5 ± 65.2	146.5 ± 55.1	0.620	MWU
Hospital stay (days)	11.97 ± 5.76	8.71 ± 4.48	0.017	MWU
ICU stay (days)	3.29 ± 4.22	1.82 ± 3.94	0.104	MWU

Values are mean ± SD. Sarcopenia defined as sex-specific bottom-tertile psoas CSA. Welch, Welch’s *t*-test (for approximately normally distributed variables); MWU, Mann–Whitney U test (for variables departing from normality). Hb, haemoglobin; pRBC, packed red blood cell; ICU, intensive care unit.

**Table 4:** Multivariable linear regression for operative time (N = 48).

Predictor	B	SE	95% CI	*p*	VIF	HC3 p
Subcutaneous fat CSA (per cm^2^)	+0.25	0.08	+0.09 to +0.41	0.004	1.10	0.068
Modified Stoppa (vs. KL)	+65.43	18.24	+28.6 to +102.3	0.001	1.17	0.024
Ilioinguinal (vs. KL)	+10.61	24.46	−38.8 to +60.0	0.667	1.11	0.539
Age (per year)	−0.34	0.48	−1.32 to +0.63	0.481	1.06	0.512
Complex fracture (vs. elementary)	−21.36	18.33	−58.4 to +15.7	0.251	1.18	0.325
Sarcopenia (vs. non-sarcopenic)	−12.66	17.98	−48.96 to +23.65	0.485	1.26	0.384

Reference categories: Kocher–Langenbeck (KL) for surgical approach; elementary fracture pattern; non-sarcopenic. Model fit: F(6, 41) = 3.71, *p* = 0.005; R^2^ = 0.352; adjusted R^2^ = 0.257. VIF, variance inflation factor (all values 1.06–1.26, indicating negligible multicollinearity). HC3 p, *p*-value from the model re-estimated with heteroscedasticity-consistent (HC3) standard errors as a sensitivity analysis.

## Data Availability

The data presented in this study are available from the corresponding author upon reasonable request, subject to institutional regulations.
